# Intestinal parasite infection in non-human primates from The Gambia, West Africa, and their relationship to human activity

**DOI:** 10.1017/S0031182025000514

**Published:** 2025-04

**Authors:** Richard S. Bradbury, Ashley R. Olson, Sarah Sapp, Indu S. Panicker, Ebenezer Foster-Nyarko, Yvonne Qvarnstrom, Martin Antonio, Mawdo Jallow, Jennifer Danzy Cramer

**Affiliations:** 1 Institute of Innovation, Science and Sustainability, Federation University Australia, Melbourne, VIC, Australia; 2 Division of Parasitic Diseases and Malaria, Centers for Disease Control and Prevention, Atlanta, GA, USA; 3 Faculty of Infectious and Tropical Diseases, London School of Hygiene and Tropical Medicine, London, UK; 4 Medical Research Council Unit, London School of Hygiene and Tropical Medicine, Fajara, Banjul, Gambia; 5 Department of Parks and Wildlife Management, Abuko Nature Reserve, Abuko, Banjul, Gambia; 6 Department of Human Resources, University of Wisconsin-Milwaukee, Milwaukee, WI, USA

**Keywords:** Africa, climate, environment, Gambia, helminths, one-health, primates, protozoa, zoonoses

## Abstract

In many areas of The Gambia, West Africa, population crowding in a degraded environment has forced close interactions of diurnal primate species with humans. We assessed intestinal parasitic infection prevalence and diversity in 4 diurnal non-human primate (NHP) species, *Chlorocebus sabaeus, Erythrocebus patas, Papio papio* and *Piliocolobus badius* across 13 sampling sites. The effect of human activity, determined by the human activity index, and NHP group size on parasite richness was assessed using a generalized linear mixed model (GLMM). The most common protozoa identified were *Entamoeba coli* (30%) and *Iodamoeba buetschlii* (25%). The most common helminths were *Strongyloides fuelleborni* (11%), *Oesophagostomum* spp. (9%) and *Trichuris trichiura* (9%). Two of six (6%) *Cyclospora* spp. infections detected sequenced as *Cyclospora cercopitheci* (both in *C. sabaeus*). The more arboreal *P. badius* trended towards a lower prevalence of intestinal parasites, although this was not statistically significant (χ^2^ *P* = 0.105). Human activity or group size did not have any significant effect on parasite richness for *P. badius* (*P* = 0.161 and *P* = 0.603) or *P. papio* (*P* = 0.817 and *P* = 0.607, respectively). There were insufficient observations to fit a GLMM to *E. patas* or *C. sabaeus*. Our reports present the richness and diversity of intestinal parasites in 4 diurnal NHPs in The Gambia, West Africa. Despite desertification and habitat loss, our results indicate that the prevalence and diversity of intestinal parasites in Gambian NHPs are seemingly unaffected by human activity. Further investigation with a larger dataset is required to better elucidate these findings.

## Graphical abstract

**Figure:**
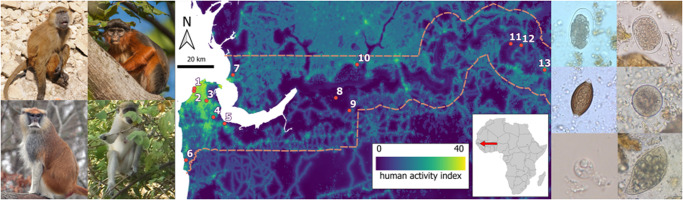

## Introduction

Non-human primates (NHPs) share many intestinal parasites with humans, and cross-species exchange of parasitic infections between NHPs and humans has been demonstrated in shared Central African tropical forest ecosystems (Medkour *et al*., 2020). Under environmental pressure, NHPs may acquire and act as reservoirs for human parasitic infection, with a flow of interspecies infection occurring. This is specifically the case for the cross-species exchange of environmentally acquired intestinal parasites, such as soil-transmitted helminths (STH) and waterborne intestinal protozoa and helminths. Such exchange has been demonstrated for the waterborne helminth *Schistosoma mansoni* infections (Kebede *et al*., 2020; Ketzis *et al*., 2020) and the STH *Strongyloides fuelleborni* (Janwan *et al*., 2020), *Ternidens deminutus* (Bradbury, 2019), *Oesophagostomum* spp. (Sirima *et al*. 2021), *Necator gorillae* (Hasegawa *et al*., 2014; Pafčo *et al*., 2019) and some sub-clades of *Trichuris trichiura* (Rivero *et al*., 2021).

In areas of West Africa where significant deforestation has occurred, there is increasing pressure on NHP populations to reside near human settlements. There has been significant recent work on the richness and diversity of intestinal parasites of NHPs in West Africa, specifically in Senegal (Medkour *et al*., 2020; N’da *et al*., 2020, 2022), Cote d’Ivoire and Sierra Leone (Köster *et al*., 2022). However, there is a paucity of information from The Gambia, a country in this region nestled within Senegal and particularly affected by desertification. The Gambia lost almost 100 000 hectares of land to desertification between 1998 and 2009 (Food and Agriculture Organization, 2021). While forest management systems are now in place, such reduction and fragmentation of habitat has been shown to place increased pressure on African NHP populations and places many groups into closer contact with humans (Bloomfield *et al*., 2020). Prior work in Uganda, East Africa (Gillespie *et al*., 2005; Gillespie and Chapman, 2008; Zommers *et al*., 2013) has demonstrated that the fragmentation of forests leads to an increase in parasite richness and diversity in resident NHPs. The combination of closer contact between humans and NHPs in shared environments and increased parasite richness and diversity in the NHP population presents a potential risk to human health (Wallis and Lee, 1999; Chapman and Peres, 2001; Chapman *et al*., 2005; Hopkins and Nunn, 2007; Devaux *et al*., 2019, 2019; Bloomfield *et al*., 2020).

The only previous studies to analyse the impact of human activity on the richness and diversity of intestinal parasitic fauna in NHPs were conducted in a lush river forest of Kenya in East Africa (Mbora and McPeek, 2009) and a variety of climates in South Africa (Gaetano *et al*., 2014). These are geographically and environmentally quite different from the Sahelian environment of The Gambia. This study serves to provide baseline information about the prevalence of intestinal helminths and protozoa among diurnal primate species found in The Gambia and determines the association of richness and diversity of these parasites with closeness to human activity.

## Methods

### Sample and data collection

**Figure 1.**
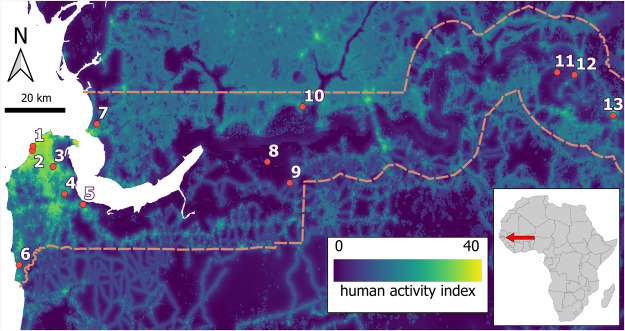
Heat map of human activity within The Gambia (map becomes lighter in colour with increased human activity), with the location of the 13 study sites superimposed (1, Senegambia; 2 Bijilo Park; 3, Abuko; 4, Makasutu; 5, Pirang; 6, Kartong; 7, Niumi; 8, Kiang-West; 9, Kiang-West HQ; 10, dia Fula; 11, River Gambia; 12, Touba; 13, Janjanbureh). The dashed line represents the political border between The Gambia and Senegal.

We collected fresh faecal samples from 4 diurnal monkey species (*Chlorocebus sabaeus, Erythrocebus patas, Papio papio* and *Piliocolobus badius*) spread across 13 sampling sites (Senegambia, Bijilo Park, Abuko, Makasutu, Pirang, Kartong, Niumi, Kiang-West, Kiang-West HQ, dia Fula, River Gambia, Touba and Janjanbureh) in The Gambia with a mix of Aw-Savannah, BSh – Arid hot steppe and localized Af – Tropical rainforest climate Köppen−Geiger Climate Classifications (Peel *et al*., 2007) and diverse surrounding physical environment (Table 1) and degree of human activity (Figure 1). Sampling was undertaken during the early onset of the wet season in June 2017, June 2018 and June 2019.

**Table 1.** Site numbers, site names, position, Köppen–Geiger climate classification and written description of the immediate environment of the 13 sites samples were collected from in this study

Site no.	Site name	Longitude	Latitude	Köppen–Geiger climate classification	Description
1	Senegambia	13.44456	−16.7244	Aw – Savannah	Beach side hotel in a densely populated urban environment.
2	Bijilo Park	13.43278	−16.7264	Aw – Savannah	Species-rich 0.5 km^2^ densely forested national park surrounded by a densely populated urban environment on 3 sides. Western side borders a beach.
3	Abuko	13.38903	−16.6542	Aw – Savannah	Species-rich 1.1 km^2^ densely forested national park surrounded by a densely populated urban environment on 3 sides. Narrow North-East side opens to a wildlife corridor leading into the Gambia river delta.
4	Makasutu	13.31481	−16.6124	Aw – Savannah	Species-rich 10.0 km^2^ privately owned forest reserve and eco-tourism centre including tributaries of the Gambia river. Surrounded by a densely populated urban environment on 3 sides. Eastern side opens to the Gambia river delta.
5	Pirang	13.286	−16.5488	Aw – Savannah	Species-rich 0.6 km^2^ dense gallery forest national park and Eco-tourism Community Project. Northern side opens to the Gambia river delta with mangroves and salt flats. Western side opens to village vegetable gardens, Eastern side to agricultural land and small communities. Southern side to a small community of people in the South-East
6	Kartong	13.12113	−16.7577	Aw – Savannah	Coastal village region in southern Gambia, bordering Senegal. Open forest, village crops and small communities. One of the southernmost parts of The Gambia, it receives more rainfall than other parts of the country.
7	Niumi	13.50542	−16.5113	Aw – Savannah	Sparsely forested semi-arid coastal region on the North bank of the Gambia river. Densely populated on its Southern border, moderately populated to the West and North. Eastern border opens out onto mangroves and the coastlines.
8	Kiang-West	13.40209	−15.9297	Aw – Savannah	Agricultural land bordering a densely forested National Park and tributary of the Gambia river to the North, East and West. Agricultural land to the South. Sparsely populated.
9	Kiang-West HQ	13.34478	−15.8515	BSh – Arid hot steppe	Centre of a 115 km^2^ sparsely populated National Park. Dry deciduous woodland National Park.
10	dia Fula	13.55217	−15.8168	BSh – Arid hot steppe	Field station in the North of the Gambia river with agricultural land to the West, open sparsely forested savannah to the North, East and South.
11	River Gambia	13.64598	−14.9586	BSh – Arid hot steppe, but site in a localized Af – Tropical rainforest climate	Riparian tropical rainforest ecology field centre and eco-tourism retreat adjoining the River Gambia within a 5.85 km^2^ national park.
12	Touba	13.63917	−14.8994	BSh – Arid hot steppe, but site in a localized Af – Tropical rainforest climate	Densely forested region adjoining the Gambia river. Small communities to the East and South.
13	Janjanbureh	13.52778	−14.7644	BSh – Arid hot steppe adjoining Aw – Savannah	Agricultural land with adjoining scrubland before a large (127 000 people) population centre to the North. Tributary of the Gambia river to the South.

To minimize the risk of duplicate sampling from a single individual, single-day troop follows were conducted by a qualified primatologist and wildlife officers. These monkey species are quite morphologically distinct, as is their faeces composition; thus, it was easy to clearly distinguish between stools from the different monkey species.

Metadata for individuals and sites was collected as follows. When possible, the sex and age class (infant, juvenile, subadult and adult) of the individual were also noted, along with GPS coordinates for each troop location, which were collected using a Garmin eTrex 10 handheld navigator. The group size for each NHP species was determined by visual inspection. Endangered animal categories were sourced from the International Union for the Conservation of Nature (IUCN)’s Red List (International Union for the Conservation of Nature, 2021).

Sampling was conducted during morning hours, beginning when monkeys were at sleeping sites from the previous night through the first 3–4 h of departing their sleeping sites. Faecal samples were collected using fresh wooden spatulas or plastic spatula inside the collection tubes. Where possible, the whole faecal sample was collected, or a portion of the centre of the faecal sample was collected, taking care not to collect portions of the stool sample contaminated by urine or environmental debris. Faecal samples were immediately stored in fixative/reagents. In 2017, samples were only stored in Total-Fix® (Medical Chemical Company, Torrance, CA). In 2018 and 2019, samples were collected in both Total-Fix® and 10% formalin (Medical Chemical Company, Torrance, CA). All the samples were stored at ambient temperature (20 to 25 °C) prior to analysis.

### Human activity index

Each site was assigned a human activity index score using the 2019 global human footprint map for terrestrial environments by Gassert *et al*. (2023). For this map, the index was calculated at a 100 m^2^ resolution and can range from 0 to 50, a higher score indicative of a greater human activity. Within The Gambia, human activity scores ranged from 3.2 to 41.1. To account for the range of values an individual NHP experiences within its home range, we used the mean human activity index calculated from all grid cells within a 2.5 km radius of each sample site for all analyses.

### Sample shipment

Total-Fix® preserved samples were shipped at ambient temperature to the Centers for Disease Control and Prevention (CDC) in the United States. These did not require a U.S. Department of Agriculture import permit as they had been specifically treated and rendered non-infectious. The 10% formalin preserved samples were shipped at ambient temperature to Federation University in Australia. An Australian Department of Agriculture, Fisheries and Forestry (DAFF) import permit was not required as these samples have been preserved and fixed correctly by a department-approved method (10% formalin; reference DAFF BICON case: preserved and fixed animal and human specimens, effective: 07 January 2021).

### Laboratory processing

The entire Total-Fix® or 10% formalin preserved samples were homogenized by shaking, then a 2 mL aliquot was diluted with 9 mL 0.85% saline and filtered through a 1 mm wire mesh, followed by centrifugation at 500 *g* for 5 min.

#### Wheatley’s trichrome stain

The packed faecal deposit was used to prepare smears for staining by mixing 5:1 with Mayers albumin (Meridian Biosciences, Cincinnati, OH) followed by smearing onto the surface of a microscope slide before being allowed to dry in ambient air at room temperature. The dried faeces and Mayer’s albumin smears were fixed in methanol for 5 min prior to staining using Wheatley’s trichrome stain (Garcia, 2009). This was followed by examination for protozoa under ×1000 oil immersion magnification.

#### Cyclospora detection by autofluorescence

A wet mount of the packed faecal deposit was made in one drop of 0.85% saline, a 18 × 18 mm coverslip placed on top and the entire coverslip scanned under ×400 magnification using ultraviolet microscopy at wavelength 350 nm to identify auto-fluorescing oocysts of *Cyclospora* spp.

#### Formalin ethyl-acetate concentration

The remaining packed faecal samples were corrected to a volume of 0.5 *g*, followed by resuspension in 9 mL of 10% formalin. The resuspended formalin solution was allowed to diffuse into the faecal matter for at least 60 min. Following this, parasite concentration of formalin-resuspended stool was performed using the formalin ethyl-acetate concentration (FEC) method (Garcia, 2009). Two 0.85% saline wet mounts of the resultant FEC deposit were prepared using 18 × 18 mm coverslips and both were scanned by light microscopy under ×100 magnification for helminth eggs and ×400 magnification for protozoa cysts and trophozoites.

#### Cyclospora *18S rRNA gene sequencing*

Total-Fix® preserved aliquots of samples that tested positive for *Cyclospora* spp. by microscopy were subjected to PCR and sequencing of the *Cyclospora* 18S rRNA gene for species identification. We utilized the UNEX-buffer method for total genomic DNA extraction (Qvarnstrom *et al*., 2018). A portion of the 18S rRNA gene was amplified using PCR primers CRYPTOFL and cycR2 (da Silva *et al*., 2003; Eberhard *et al*., 2014). PCR products were Sanger sequenced using the BigDye V3.1 chemistry on an ABI Prism 3100 sequence analyser (Life Technologies).

### Statistical analysis

A χ^2^ test in Microsoft Excel (version 2208) was used to compare the prevalence of any intestinal parasite, intestinal protozoa and STH infection in each NHP species sampled. All other statistical analyses were performed using the R software environment (version 4.1.2). For the 2 most surveyed species of NHP, *P. papio* (*n* = 33) and *P. badius* (*n* = 28), we fitted a generalized linear mixed model (GLMM) to identify the effect of human activity and group size on parasite richness within an individual using the *glmer* function in the *lme4* package (Bates *et al*., 2015). We fitted each GLMM with a Poisson error distribution and a logarithmic link function, which is appropriate when the response variable is count data (Zuur *et al*., 2009). The human activity index and group size were included in each model as fixed effects, whereas sample site was included as a random effect to account for observations at each site not being statistically independent (Zuur *et al*., 2009). Given that collinearity among predictor variables can influence coefficient estimates and statistical power in GLMM, models were inspected for multicollinearity by measuring the variance inflation factor (VIF) using the *vif* function in the *car* package (Fox and Weisberg, 2019). We also tested for spatial autocorrelation within our models by calculating Moran’s *I* using residuals grouped by sample site using the *testSpatialAutocorrelation* in the *DHARMa* package (Hartig, 2020). There was no evidence of strong collinearity among predictor variables (VIF < 5.0 in all cases) or spatial autocorrelation in model residuals (Moran’s *I* between −1 and 1 in all cases), and thus, we proceeded without further consideration of either phenomenon. Residual vs. fitted value plots were inspected to ensure model residuals did not violate the statistical assumptions required for parametric tests. For each model, we report the parameter coefficients, *z*-scores and associated *P*-values for human activity and group size.

## Results

### NHP distribution across sampling sites

**Figure 2.**
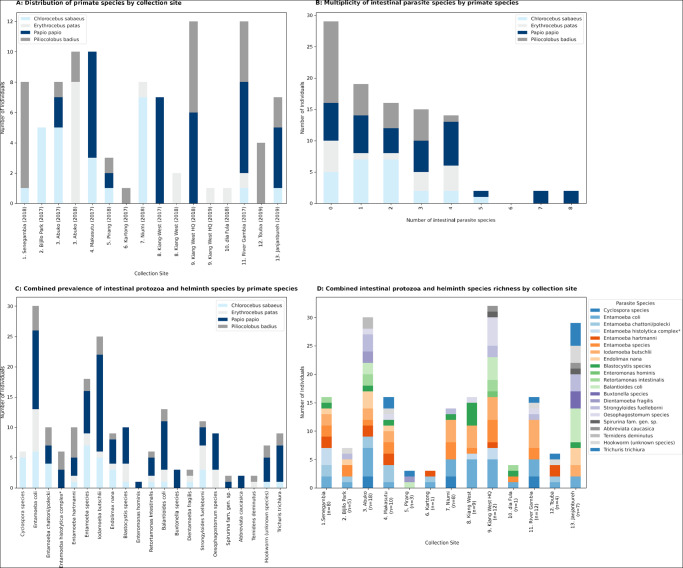
Results of faecal samples collection and parasite screening of four diurnal non-human primate (NHP) species conducted in The Gambia, West Africa, in June 2017, June 2018, and June 2019. (A) Distribution of specimens collected at each sampling site; (B) Multiplicity of infection in NHP species; (C) Prevalence of intestinal protozoa and helminth species in NHP species; (D) Combined intestinal protozoa and helminth richness by study site. *The *Entamoeba histolytica* complex includes *Entamoeba nuttali*, *E. histolytica*, *E. dispar*, *E. moshkovski* and *E. bangladeshi*, which cannot be differentiated by microscopy alone.

A total of 99 NHPs from all 4 diurnal species indigenous to The Gambia were sampled over the sampling period as follows: 43 in 2017, 44 in 2018 and 12 in 2019. Not all NHP species were observed at each sample site. We observed and collected samples from *C. sabaeus* (IUCN species of least concern; *n* = 24) at 8 sites, *E. patas* (IUCN near threatened species; *n* = 14) at 6 sites, *P. papio* (IUCN near threatened species; *n* = 33) at 8 sites and *P. badius* (IUCN endangered species; *n* = 28) at 9 sites (Figure 2a).

### Prevalence and diversity of intestinal helminths and protozoa

**Figure 3.**
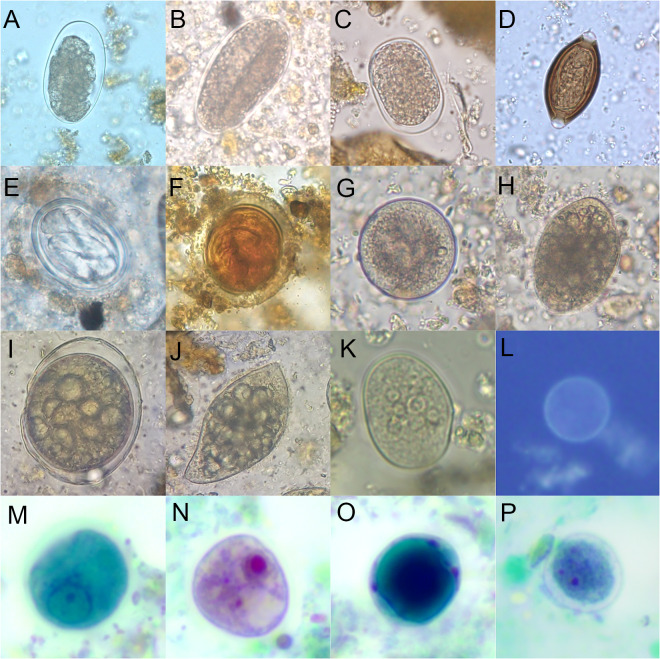
Selected intestinal parasites identified in four diurnal non-human primate species specimens collected at each sampling site in The Gambia, June 2017 June 2018, and June 2019. In wet preparation microscopy; (A) hookworm egg, (B) *Oesophagostomum* egg, (C) *Strongyloides fuelleborni fuelleborni* egg, (D) *Trichuris trichiura* egg, (E) *Spirurina* fam. gen. sp. egg type 1, (F) *Spirurina* fam. gen. sp. egg type 2, (G) *Balantioides coli* cyst, (H) *Balantioides coli* trophozoite; (I) *Buxtonella* sp. cyst, (J) *Buxtonella* sp. trophozoite, (K) *Entamoeba coli* cyst; (L) autofluorescent *Cyclospora* sp. oocyst at wavelength 350 nm, and, in trichrome stain; (M) *Entamoeba chattoni/polecki* cyst, (N) *Iodamoeba buetschlii* cyst, (O) *Blastocystis* sp. trophozoite and, (P) *Endolimax nana* cyst.

We observed 21 intestinal parasite species from at least 17 separate genera, one genus of coccidia (*Cyclospora* spp.), 4 species of *Entamoeba*, 2 ciliate protozoa (*Balantioides coli* and *Buxtonella* sp.) and 7 other genera of protozoa. In 18 cases, the species of *Entamoeba* could not be determined due to morphological ambiguity. At least 5 genera of STH (including hookworms, which may represent multiple genera) were identified, as well as 2 insect-intermediate-host-transmitted spirurid nematode genera (*Spirurina* fam. gen. sp.) (Figure 3).

At least one intestinal parasite was detected in 82% of *P. papio*, 79% of *C. sabaeus*, 64% of *E. patus* and 54% of *P. badius*, with a mean average intestinal parasite prevalence of 71% across all NHP species examined (Figure 2b). The difference in overall parasite prevalence between the 4 NHP species was not statistically significant (χ^2^ *P* = 0.105). Regarding the average richness of parasitic infections, *P. papio* had the highest mean average richness for individuals (mean average = 2.8, median = 3); followed by *E. patas* (mean average = 2.0, median = 1.5); then *C. sabaeus* (mean average = 1.7, median = 1.5) and *P. badius* (mean average = 1.1, median = 1.0). Guinea baboons (*P. papio*) were most likely to harbour more than one intestinal parasite (Figure 2b).

The prevalence of intestinal protozoan infection was 82% in *P. Papio*, 75% in *C. sabaeus*, 64% in *E. patus*, 50% in *P. badius* and 69% across all NHP species examined. These differences in protozoan prevalence by species were not statistically significant (χ^2^ *P* = 0.051). The most common species of intestinal protozoa were *Entamoeba coli* (30%), followed by *I. buetschlii* (25%) (Figure 2c). Five (83%) *Cyclospora* infections identified were in *C. sabaeus*. A single *E. patas* was also identified (Figure 2c). *Cyclospora* 18S rRNA PCR was performed on 6 positive samples. Two samples (both from *C. sabaeus* at site 7) were positive by PCR and generated DNA sequences almost identical (one base pair difference in 1014 base pairs total; GenBank accession number OR699281) to *Cyclospora cercopitheci* 18S rRNA.

The prevalence of STH infection was 39% in *P. papio*, 43% in *E. patas*, 21% in *C. sabaeus*, 14% in *P. badius* and 28% across all NHP species examined; however, these differences were not statistically significant (χ^2^ *P* = 0.078). The most prevalent species of helminth was *Strongyloides fuelleborni* subsp. *fuelleborni* (11%), then *Oesophagostomum* spp. (9%), *T. trichiura* (9%) and hookworms (7%). Five of eight *Oesophagostomum* spp. infections (60%) were from a small geographic region of Kiang West province (Figure 1). The hookworms, *T. trichiura* and *S. f. fuelleborni* infections showed no apparent geographic clustering (Figure 2d).

### The effect of human activity and group size on parasite richness

The results of the GLMM for *P. badius* indicated that there was no significant association with human activity (*β* = 0.604, *z* = 1.401, *P* = 0.161) or group size (*β* = 0.158, *z* = 0.520, *P* = 0.603) on parasite richness. Similarly, the GLMM for *P. papio* indicated that there was no significant effect of human activity (*β* = −0.048, *z* = −0.232, *P* = 0.817) or group size (*β* = −0.098, *z* = −0.514, *P* = 0.607) on parasite richness in individuals. There were insufficient observations from insufficient sites to fit a GLMM to *E. patas* or *C. sabaeus*.

## Discussion

This study represents the first survey of such parasites in NHPs from The Gambia, and 1 of only 6 surveys performed in the semi-arid Sudanian environmental zone of West Africa (McGrew *et al*., 1989; Howells *et al*., 2011; Joshua *et al*., 2020; Medkour *et al*., 2020; N’da *et al*., 2020). Our results reveal a greater diversity of parasite species than many others from the Sudanian climate region, with Gambian NHPs having a richness of intestinal parasite fauna only matched by chimpanzees in the nearby south-eastern region of Senegal (McGrew *et al*., 1989; Howells *et al*., 2011). This may reflect increased habitat pressure on Gambian NHPs in comparison to those elsewhere. The Gambia lost almost 100 000 hectares of land between 1998 and 2009 to desertification (Food and Agriculture Organization, 2021), and while forest management strategies are now in place, this reduction in habitat may have placed increased parasitic disease pressure on the Gambian NHP population.

Habitat loss and an increasing human population in this region have led to greater contact between NHPs and humans in some regions of West Africa and elsewhere (Chapman and Peres, 2001). Furthermore, primate populations have been restricted to tightly confined areas of remnant habitat (Wallis and Lee, 1999; Chapman and Peres, 2001). Both factors increase stress on individual NHPs, reduce population size and expose them to a higher risk of disease acquisition and transmission between humans and animals (Wallis and Lee, 1999; Chapman *et al*., 2005). Both humans and NHPs are likely to share water sources and are thereby exposed to the same gastrointestinal protozoa. Similarly, humans and NHPs will be exposed to the same infective stages of STH in the soil. For such parasites, it is deemed likely that such cross-species parasite transmission will occur within a given geographical environment (Chapman *et al*., 2005). However, our study did not observe any significant increase in the richness of intestinal parasite species in *P. badius* and *P. papio* in sites with higher human activity indices than in sites with lower human activity. *Erythrocebus patas* and *C. sabaeus* were not assessed due to insufficient sample size. Gaetano *et al*. (2014) similarly reported no effect of anthropogenic activity (as determined by reference to a global human population density grid similar to the human activity index employed in our study) on intestinal parasitism in 78 South African vervet monkeys (*Chlorocebus aethiops*). In contrast, Mbora and McPeek (2009) compared human activity, measured by the basal area of cut stems at a given site, with the richness and diversity of intestinal parasites in 2 species of NHP within the Tana River Primate National Reserve in Kenya. In this study, Tana River red colobus (*Piliocolobus rufomitratus*) (*n* = 150) and mangabeys (*Cercocebus galeritus*) (*n* = 81) demonstrated increased parasite prevalence and richness associated with greater population density and crowding due to decreased and fragmented habitat consequent to human activity (Mbora and McPeek, 2009). It remains possible that the much larger sample size employed in this Kenya study is responsible for the variation in findings of their work when compared to that of our work and that of Gaetano *et al*. (2014).

The greatest richness in parasite infection was observed in *P. papio*, consistent with increased richness of parasites in *Papio* spp. in other studies (Frias and MacIntosh, 2020). Being terrestrial, highly mobile and more likely to be in close proximity to humans during foraging are considered favourable factors leading to higher parasite prevalence in Guinea baboons from Senegal (N’da *et al*., 2022). We consider this to be the likely reason for the increased parasite richness also observed in Gambian *P. papio* Guinea baboons.

Gambian *P. badius* showed a lower prevalence of overall intestinal parasite, intestinal protozoan and STH infections when compared to the other NHP species sampled, although this did not reach significance. This is consistent with Mbora and McPeek (2009), which also found a higher prevalence of parasites in terrestrial mangabeys than in arboreal Tana River red colobus monkeys, but unlike our data, their findings did reach significance (*P* < 0.01). Mbora and McPeek (2009) postulated that NHP species behaviour leads to these findings, and we agree. The more arboreal nature of *P. badius* may contribute to this phenomenon. Observational studies in The Gambia have noted that *P. badius* monkeys rarely descend to the ground to obtain water, and only do so during the dry season (Hillyer *et al*., 2015). This species mostly obtains water from leaves, collections in tree trunk depressions, or by licking their bodies after rain (Hillyer *et al*., 2015). The primary use of relatively ‘cleaner’ water sources by *P. badius*, and their aversion to terrestrial activity, likely reduces the opportunities for exposure to waterborne protozoa and STH from both humans and other NHP species. While the lower intestinal parasite prevalence observed in *P. badius* was not statistically significant in our study, additional investigation with larger sample sizes would better elucidate this hypothesis.

This study provides the first identification of *Cyclospora* infection in *C. sabaeus* and *E. patas* hosts. Since the morphology of all primate *Cyclospora* species is the same or very similar, the determination of the various *Cyclospora* species requires sequencing. Two of these infections (occurring in *C. sabaeus* hosts) were possible to identify by sequencing as *C. cercopitheci*. Originally, these were identified in *C. aethiops* from Ethiopia (Eberhard *et al*., 1999) and later Kenya (Eberhard *et al*., 2001). This report expands both the known hosts and the geographical range of this parasite, as all prior reports were restricted to East Africa (Eberhard *et al*., 1999, 2001; Lopez *et al*., 1999; Li *et al*., 2011), or much further south in Equatorial Guinea (Eberhard *et al*., 2014).

Wild NHP stool is difficult to obtain. The use of only one stool per individual likely reduced the recovery of intestinal parasites in this study (Garcia, 2009) but was unavoidable. The choice of preservative was changed during the study due to Australian biosecurity importation rules, and this may have had a minor influence on the recovery rate of low-intensity parasite infections. This may have affected the findings of samples tested between 2017 and 2018–2019. A larger study, incorporating more individuals and collection of samples from both humans and monkeys in the same area, with associated genotyping, is indicated.

In summary, our study provides insight into the richness and diversity of intestinal parasites in NHPs from The Gambia, West Africa. Despite increased contact between NHPs and humans due to desertification, our results suggest that the richly diverse population of parasites in these diurnal monkeys is not influenced by human activity or group size in this setting; further investigation with a larger dataset is required to better elucidate these findings.

## References

[ref1] Bates D, Mächler M, Bolker B and Walker S (2015) Fitting linear mixed-effects models using lme4. *Journal of Statistical Software* 67, 1–48. doi:10.18637/jss.v067.i01

[ref2] Bloomfield LS, McIntosh TL and Lambin EF (2020) Habitat fragmentation, livelihood behaviors, and contact between people and nonhuman primates in Africa. *Landscape Ecology* 35, 985–1000. doi:10.1007/s10980-020-00995-w

[ref3] Bradbury RS (2019) *Ternidens deminutus* revisited: A review of human infections with the false hookworm. *Tropical Medicine and Infectious Disease* 4(3), 106. doi:10.3390/tropicalmed4030106 31323820 PMC6789545

[ref4] Chapman CA, Gillespie TR and Goldburg TL (2005) Primates and the ecology of their infectious diseases: How will anthropogenic change affect host-parasite interactions? *Evolutionary Anthropology* 14, 134–144. doi:10.1002/evan.20068

[ref5] Chapman CA and Peres CA (2001) Primate conservation in the new millennium: The role of scientists. *Evolutionary Anthropology* 10, 16–33. doi:10.1002/1520-6505(2001)10:1<16::AID-EVAN1010>3.0.CO;2-O

[ref6] da Silva AJ, Cacciò S, Williams C, Won KY, Nace EK, Whittier C, Pieniazek NJ and Eberhard ML (2003) Molecular and morphologic characterization of a *Cryptosporidium* genotype identified in lemurs. *Veterinary Parasitology* 111, 297–307. doi:10.1016/s0304-4017(02)00384-9 12559709

[ref7] Devaux CA, Mediannikov O, Medkour H and Raoult D (2019) Infectious disease risk across the growing human-non human primate interface: A review of the evidence. *Frontiers in Public Health* 7, 305. doi:10.3389/fpubh.2019.00305 31828053 PMC6849485

[ref8] Eberhard ML, da Silva AJ, Lilley BG and Pieniazek NJ (1999) Morphologic and molecular characterization of new *Cyclospora* species from Ethiopian monkeys: *C. cercopitheci* sp. n., *C. colobi* sp. n., and *C. papionis* sp. n. *Emerging Infectious Diseases* 5, 651. doi:10.3201/eid0505.990506 10511521 PMC2627716

[ref9] Eberhard ML, Njenga MN, DaSilva AJ, Owino D, Nace EK, Won KY and Mwenda JM (2001) A survey for *Cyclospora* spp. in Kenyan primates, with some notes on its biology. *Journal of Parasitology* 87, 1394–1397. doi:10.1645/0022-3395(2001)087[1394:ASFCSI]2.0.CO;2 11780827

[ref10] Eberhard ML, Owens JR, Bishop HS, de Almeida ME and da Silva AJ (2014) *Cyclospora* spp. in drills, Bioko Island, Equatorial Guinea. *Emerging Infectious Diseases* 20, 510–511. doi:10.3201/eid2003.131368 24565509 PMC3944859

[ref11] Food and Agriculture Organization (2021) *Action Against Desertification – The Gambia*. Retrieved from Food and Agriculture Organization of the United Nations website. Available at http://www.fao.org/in-action/action-against-desertification/countries/africa/gambia/en/ (accessed 17 May 2021).

[ref12] Fox J and Weisberg S (2019) *An R Companion to Applied Regression*, 3rd edn. Thousand Oaks, CA: Sage.

[ref13] Frias L and MacIntosh AJ (2020) Global diversity and distribution of soil-transmitted helminths in monkeys. In Knauf S and Jones-Engel L (eds), *Neglected Diseases in Monkeys*. New York: Springer Cham, pp. 291–322.

[ref14] Gaetano TJ, Danzy J, Mtshali MS, Theron N, Schmitt CA, Grobler JP, Freimer N and Turner TR (2014) Mapping correlates of parasitism in wild South African vervet monkeys (*Chlorocebus aethiops*). *South African Journal of Wildlife Research* 44, 56–70. doi:10.3957/056.044.0105

[ref15] Garcia LS (2009) *Practical Guide to Diagnostic Parasitology*, 2nd edn. Washington, D.C.: American Society for Microbiology.

[ref16] Gassert F, Ventner O, Watson JE, Brumby SP, Mazzariello JC, Atkinson SC and Hyde S (2023) An operational approach to near real time global high resolution mapping of the terrestrial human footprint. *Frontiers in Remote Sensing* 4, 1130896. doi:10.3389/frsen.2023.1130896

[ref17] Gillespie TR and Chapman CA (2008) Forest fragmentation, the decline of an endangered primate, and changes in host–parasite interactions relative to an unfragmented forest. *American Journal of Primatology* 70, 222–230. doi:10.1002/ajp.20475 17879941

[ref18] Gillespie TR, Chapman CA and Greiner EC (2005) Effects of logging on gastrointestinal parasite infections and infection risk in African primates. *Journal of Applied Ecology* 42, 699–707. doi:10.1111/j.1365-2664.2005.01049.x

[ref19] Hartig F (2020) *DHARMa: Residual Diagnostics for Hierarchical (Multi-level/mixed) Regression Models*. R package v. 0.3-3.

[ref20] Hasegawa H, Modrý D, Kitagawa M, Shutt KA, Todd A, Kalousová B, Profousová I and Petrželková KJ (2014) Humans and great apes cohabiting the forest ecosystem in Central African Republic Harbour the same hookworms. *PLoS Neglected Tropical Diseases* 8, e2715. doi:10.1371/journal.pntd.0002715 24651493 PMC3961186

[ref21] Hillyer AP, Armstrong R and Korstjens AH (2015) Dry season drinking from terrestrial man-made watering holes in arboreal wild Temminck’s red colobus, The Gambia. *Primate Biology* 2, 21–24. doi:10.5194/pb-2-21-2015

[ref22] Hopkins ME and Nunn CL (2007) A global gap analysis of infectious agents in wild primates. *Diversity and Distributions* 5, 561–572. doi:10.1111/j.1472-4642.2007.00364.x

[ref23] Howells ME, Pruetz J and Gillespie TR (2011) Patterns of gastro‐intestinal parasites and commensals as an index of population and ecosystem health: The case of sympatric Western chimpanzees (*Pan troglodytes verus*) and Guinea baboons (*Papio hamadryas papio*) at Fongoli, Senegal. *American Journal of Primatology* 73, 173–179. doi:10.1002/ajp.20884 20853397

[ref24] International Union for the Conservation of Nature (2021) *Red List*. Retrieved IUCN Redlist website. Available at https://www.iucnredlist.org (accessed 17 May 2021).

[ref25] Janwan P, Rodpai R, Intapan PM, Sanpool O, Tourtip S, Maleewong W and Thanchomnang T (2020) Possible transmission of *Strongyloides fuelleborni* between working Southern pig-tailed macaques (*Macaca nemestrina*) and their owners in Southern Thailand: Molecular identification and diversity. *Infection Genetics & Evolution* 85, 104516. doi:10.1016/j.meegid.2020.104516 32860989

[ref26] Joshua K, Yidawi JP, Sada A, Msheliza EG and Turaki UA (2020) Prevalence and morphotype diversity of *Trichuris* species and other soil-transmitted helminths in captive non-human primates in northern Nigeria. *Journal of Threatened Taxa* 12, 16239–16244. doi:10.11609/jott.4552.12.10.16239-16244

[ref27] Kebede T, Bech N, Allienne JF, Olivier R, Erko B and Boissier J (2020) Genetic evidence for the role of non-human primates as reservoir hosts for human schistosomiasis. *PLoS Neglected Tropical Diseases* 14, e0008538. doi:10.1371/journal.pntd.0008538 32898147 PMC7500647

[ref28] Ketzis JK, Lejeune M, Branford I, Beierschmitt A and Willingham AL (2020) Identification of *Schistosoma mansoni* infection in a nonhuman primate from St. Kitts more than 50 years after interruption of human transmission. *The American Journal of Tropical Medicine and Hygiene* 103, 2278–2281. 10.4269/ajtmh.20-0282. Epub 2020 Sep 24 32996451 PMC7695088

[ref29] Köster PC, Lapuente J, Pizarro A, Prieto-Pérez L, Pérez-Tanoira R, Dashti A, Bailo B, Muadica AS, González-Barrio D, Calero-Bernal R and Ponce-Gordo F (2022) Presence and genetic diversity of enteric protists in captive and semi-captive non-human primates in côte d’Ivoire, Sierra Leone, and Peru. *International Journal for Parasitology: Parasites and Wildlife* 17, 26–34. doi:10.1016/j.ijppaw.2021.12.004 34976722 PMC8688894

[ref30] Li W, Kiulia NM, Mwenda JM, Nyachieo A, Taylor MB, Zhang X and Xiao L (2011) *Cyclospora papionis, Cryptosporidium hominis*, and human-pathogenic *Enterocytozoon bieneusi* in captive baboons in Kenya. *Journal of Clinical Microbiology* 49, 4326–4329. doi:10.1128/JCM.05051-11 21956988 PMC3232936

[ref31] Lopez FA, Manglicmot J, Schmidt TM, Yeh C, Smith HV and Relman DA (1999) Molecular characterization of *Cyclospora*-like organisms from baboons. *The Journal of Infectious Diseases* 179, 670–676. doi:10.1086/314645 9952374

[ref32] Mbora DN and McPeek MA (2009) Host density and human activities mediate increased parasite prevalence and richness in primates threatened by habitat loss and fragmentation. *Journal of Animal Ecology* 78, 210–218. doi:10.1111/j.1365-2656.2008.01481.x 19120603

[ref33] McGrew WC, Tutin CE, Collins DA and File SK (1989) Intestinal parasites of sympatric *Pan troglodytes* and *Papio* spp. at two sites: Gombe (Tanzania) and Mt. Assirik (Senegal). *American Journal of Primatology* 17, 147–155. doi:10.1002/ajp.1350170204 31968849

[ref34] Medkour H, Amona I, Laidoudi Y, Davoust B, Bitam I, Levasseur A, Akiana J, Diatta G, Pacheco L, Gorsane S and Sokhna C (2020) Parasitic infections in African humans and non-human primates. *Pathogens* 9, 561. doi:10.3390/pathogens9070561 32664573 PMC7400533

[ref35] N’da KM, Dahourou LD, Gbati OB and Alambedji RB (2020) Diversity and prevalence of gastrointestinal parasites with zoonotic potential of Green Monkeys in Bandia Reserve in Senegal. *International Journal of One Health* 7, 65–69. doi:10.14202/IJOH.2021.65-69

[ref36] N’da KM, Dahourou LD, Ndiaye PI, Lindshield S, Gbati OB and Traore A (2022) Gastrointestinal parasites of baboons (*Papio papio*) in Niokolo-Koba National Park, Senegal. *Open Veterinary Journal* 12, 481–488. doi:10.5455/OVJ.2022.v12.i4.9 36118726 PMC9473380

[ref37] Pafčo B, Kreisinger J, Čížková D, Pšenková-Profousová I, Shutt-Phillips K, Todd A, Fuh T, Petrželková KJ and Modrý D (2019) Genetic diversity of primate strongylid nematodes: Do sympatric nonhuman primates and humans share their strongylid worms? *Molecular Ecology* 28, 4786–4797. doi:10.1111/mec.15257 31573713

[ref38] Peel MC, Finlayson BL and McMahon TA (2007) Updated world map of the Köppen-Geiger climate classification. *Hydrology and Earth System Sciences* 11, 1633–1644. doi:10.5194/hess-11-1633-2007

[ref39] Qvarnstrom Y, Benedict T, Marcet PL, Wiegand RE, Herwaldt BL and da Silva AJ (2018) Molecular detection of *Cyclospora cayetanensis* in human stool specimens using UNEX-based DNA extraction and real-time PCR. *Parasitology* 145, 865. doi:10.1017/S0031182017001925 29113617 PMC5940589

[ref40] Rivero J, Cutillas C and Callejón R (2021) *Trichuris trichiura* (Linnaeus, 1771) from human and non-human primates: Morphology, biometry, host specificity, molecular characterization, and phylogeny. *Frontiers in Veterinary Science* 9, 626120. doi:10.3389/fvets.2020.626120 PMC7934208 33681315

[ref41] Sirima C, Bizet C, Hamou H, Červená B, Lemarcis T, Esteban A, Peeters M, Mpoudi Ngole E, Mombo IM, Liégeois F and Petrželková KJ (2021) Soil-transmitted helminth infections in free-ranging non-human primates from Cameroon and Gabon. *Parasites and Vectors* 14, 1–8. doi:10.1186/s13071-021-04855-7 34225777 PMC8259424

[ref42] Wallis J and Lee DR (1999) Primate conservation: The prevention of disease transmission. *International Journal of Primatology* 20, 803–826. doi:10.1023/A:1020879700286

[ref43] Zommers Z, Macdonald DW, Johnson PJ and Gillespie TR (2013) Impact of human activities on chimpanzee ground use and parasitism (*Pan troglodytes*). *Conservation Letters* 6, 264–273. doi:10.1111/j.1755-263X.2012.00288.x

[ref44] Zuur AF, Ieno EN, Walker N, Saveliev AA and Smith GM (2009) *Mixed Effects Models and Extensions in Ecology with R*. New York: Springer.

